# U.S. Adolescents’ Diet Consumption Patterns Differ between Grocery and Convenience Stores: National Health and Nutrition Examination Survey 2011–2018

**DOI:** 10.3390/ijerph18168474

**Published:** 2021-08-11

**Authors:** Felicia J. Setiono, Navika Gangrade, Tashara M. Leak

**Affiliations:** Division of Nutritional Sciences, Savage Hall, Cornell University, Ithaca, NY 14850, USA; ng463@cornell.edu (N.G.); tml226@cornell.edu (T.M.L.)

**Keywords:** adolescents, food groups, grocery stores, convenience stores, NHANES

## Abstract

Among U.S. adolescents, foods/beverages that are store-bought (i.e., from grocery and convenience stores) are significant contributors of energy intake. However, it remains unclear (1) what foods/beverages are consumed by U.S. adolescents from grocery and convenience stores and (2) if there are differences in foods/beverages consumed by store type. Therefore, we analyzed 29,216 eating occasions from adolescents (12–19 years; *n* = 4065) in the National Health and Nutrition Examination Survey 2011–2018 to report food/beverage groups and nutrients consumed from grocery and convenience stores. Differences in food/beverage groups and nutrient densities by store type were calculated using multiple logistic and linear regressions. Adolescents were more likely to consume “Beverages” and “Snacks and Sweets” and less likely to consume “Grains”, “Protein”, “Milk and Dairy”, “Condiments and Sauces”, and “Fruits” from convenience compared to grocery stores (all *p* < 0.0025). Foods/beverages from convenience stores were higher in carbohydrates, total sugar, and added sugar and lower in protein, fat, saturated fat, sodium, and fiber than those from grocery stores (all *p* < 0.0025). In conclusion, while foods/beverages from convenience stores are more energy-dense and nutrient-poor, there is a critical need to increase the availability, accessibility, and affordability of healthier foods/beverages in both store types to encourage healthier dietary behaviors among U.S. adolescents.

## 1. Introduction

In the United States, adolescents (12–19 years) are not meeting federal dietary recommendations [1,2], and research suggests where foods/beverages are sourced from is an important social determinant of diet [3]. While there are a variety of locations that adolescents source food from, such as cafeterias, restaurants, and fast-food locations, store-bought foods (i.e., foods from grocery and convenience stores) are the largest contributors to energy [4,5]. Results from a nationally representative survey report that almost half of U.S. adolescents visit convenience stores weekly [6], though it remains unclear how often adolescents visit grocery stores.

Some studies have examined the relationship between neighborhood food environment and adolescent diet and health. Research reveals that living in proximity to convenience stores is associated with an increased risk of having lower diet quality [7] and obesity [8,9]. A study by Hager et al. (2017) found that adolescents living in areas with high access to convenience stores were more likely to consume snacks and desserts than those living in areas with low access to convenience stores [10]. Another reported that adolescents who live further from grocery stores have a lower intake of healthy foods, such as fruits [11]. Consistently, a different study concluded that adolescents with higher access to grocery stores have reduced risk of obesity [8]. Therefore, there is a need to consider explanations for the associations between store type and adolescent diet and health.

One explanation for associations between store type and diet and health may be the difference in the availability of foods and beverages in these stores. Research reveals that convenience stores have a higher availability of unhealthy, energy-dense, nutrient-poor (EDNP) foods/beverages (e.g., salty snacks, sugar-sweetened beverages) [12,13,14,15], while grocery stores generally have a higher availability of healthy, nutrient-dense foods (e.g., fruits and vegetables) [16,17,18,19]. In response, there has been substantial efforts to promote healthy, nutrient-dense foods/beverages in convenience stores, with most strategies striving to increase the availability of fruits and vegetables [20,21]. However, measurement of these strategies’ effects on the diet and health outcomes of adolescents, specifically, are limited [20,21]. There has also been some interest in promoting healthy food/beverage choices in grocery stores by implementing nutrition education sessions, offering healthy food/beverage coupons, and labeling foods/beverages by healthfulness [22]. Finally, adding new grocery stores to a community is another strategy to improve the availability of healthy foods/beverages. However, these attempts in grocery stores have yielded modest effects on diet and health outcomes in real world settings [23,24,25,26]. It is possible that these interventions are not targeting foods/beverages that are relevant to adolescents. Thus, it is important to consider and understand what foods/beverages that are consumed by adolescent come from grocery and convenience stores.

The overall aim of this study is to utilize data from U.S. adolescents participating in the National Health and Nutrition Examination Survey (NHANES) 2011–2018 to examine (1) what food/beverage groups and nutrients consumed are sourced from grocery and convenience stores and (2) the differences in consumption of these food/beverage groups and nutrients by the two food store types.

## 2. Materials and Methods

### 2.1. Study Design and Population

NHANES is a complex, multistage probability cross-sectional study of a nationally representative sample of U.S. civilian, noninstitutionalized populations conducted by the National Center for Health Statistics [27]. Data from adolescents (12–19 years) who participated in four two-year cycles from 2011–2018 were included in this analysis. Adolescents 12–17 years provided assent with parental consent, and adolescents 18–19 years provided consent. NHANES was approved by the National Center for Health Statistics Research Ethics Review Board (protocol #2011–17 for years 2011–16, protocol #2011-17 and #2018–01 for years 2017–2018). Given that this study is a secondary data analysis of NHANES data, which is publicly available and de-identified, this study was considered not human subjects research under the Cornell University institutional review board policy and does not require review [28].

### 2.2. Measures

#### 2.2.1. Sociodemographic Characteristics

The demographic characteristics of participants were obtained through an at-home interview administered by trained interviewers. Adolescents who were 12–15 years of age had a proxy to respond to questions when they were unable to answer, whereas those 16 and older answered all survey questions independently. Sex (male or female) and race/ethnicity (non-Hispanic [NH] black, NH white, Mexican American, other Hispanic, NH Asian, and other race/multi-racial) were self-reported. Age was calculated based on each participant’s birth date. Socioeconomic status was measured using household poverty to income ratio (PIR), which is calculated by dividing family income by the poverty threshold specific to survey year and family size. PIR was characterized into two categories: low income (PIR < 3.0) and high income (≥3.0) as done previously by another study [29].

#### 2.2.2. Dietary Intake

Dietary intake data was obtained through an interviewer-administered 24-h dietary recall in a private room in the Mobile Examination Center (MEC) using the USDA Automated Multiple-Pass Method, which has been shown to be a reliable and practical method for collecting dietary intake information [30]. Adolescents 12 years or older were asked to estimate the types and amounts of all foods and beverages consumed in the preceding 24-h period. A second 24-h dietary recall was collected via telephone 3 to 10 days after the interviewer-administered recall. In this study, only the first day diet information from each adolescent, as well as only those labeled as reliable entries, were included in the analysis. While a second day diet data may provide additional information on adolescents’ usual intake, the second day data is not as reliable as the first day data and is often missing. In addition, we would not have been able to characterize the food/beverage groups that are most commonly consumed from each store type (by the proportion of adolescents consuming it on a given day).

The source from which each food and beverage was obtained was also collected from a list of 29 different types of food sources. Specifically, for each food/beverage, the adolescents were asked “where did you get (this/most of the ingredients for this) food/beverage?” In this study, we included only those foods/beverages sourced from grocery/supermarket (referred to as grocery stores hereafter) and convenience stores. In the dataset, each food and beverage item were given a Food and Nutrient Database for Dietary Studies (FNDDS) code. This code was then converted into the USDA What We Eat in America (WWEIA) Food Categories [31] using the Food Patterns Equivalent Database (FPED). In this study, the WWEIA Food Categories will be referred to as food/beverage groups. We will report on the top 10 WWEIA food/beverage groups by the proportion of each food/beverage group consumed from grocery and convenience stores among all foods/beverages consumed from grocery and convenience stores. The “Water” food/beverage group (which includes tap, bottled, flavored, and enhanced water) was excluded from all analyses as this study is interested in understanding the nutritional contributions of foods/beverages consumed by adolescents.

We will also report the average energy contribution of foods/beverages from grocery and convenience stores, as well as the mean nutrient densities (grams, milligrams, or micrograms of the nutrient per 100 kcal of the food/beverage). The nutrients examined for this analysis were the macronutrients (carbohydrate, protein, fat) and dietary components of public health interest, based on the USDA Dietary Guidelines for Americans (i.e., sugar [total and added sugar], saturated fat, sodium, and fiber) [32].

### 2.3. Statistical Analyses

Survey weights, strata, and clustering variables were used to account for the complex, multistage probability sampling design used in NHANES [33,34]. Dietary data weights were used to create 8-year weights (i.e., 4 cycles) for all analyses. Continuous variables were described using weighted means and categorical variables were summarized with weighted proportions and standard errors (SE) estimated with Taylor series linearization.

Differences in the probability of food groups by food store were calculated using survey-weighted multiple logistic regression models, adjusted for sex, age, race/ethnicity, and household PIR status. Differences in nutrient information were calculated using survey-weighted multiple linear regressions, adjusted for the same covariates as above. Fitted residuals from the linear models were plotted using histograms to assess normality. If distributions were highly skewed, data was log- or square root-transformed. The significance level was set at *p* < 0.0025 based on Bonferroni correction to account for multiple hypotheses testing conducted in the study from the various regression models. The analyses were conducted using SAS Version 9.4 (Cary, NC, USA) and STATA 14 (College Station, TX, USA).

## 3. Results

The weighted proportion of foods and beverages consumed by store type across individual and household characteristics of U.S. adolescents (*n* = 4235) are presented in Table 1. The proportion of adolescents aged 16–19 years (56.8%) slightly exceeded that of those aged 12–15 (43.2%). About half of the adolescents were male (50.3%) and half were female (49.7%). The adolescents mostly identified as NH white (52.6%), followed by Mexican American (15.8%), NH black (14.0%), other Hispanic (7.4%), other race/multi-racial (5.1%), and NH Asian (5.0%). In terms of PIR status, 64.0% of adolescents were categorized as coming from low-income households (PIR < 3.0) and 36.0% as coming from high-income households (PIR ≥ 3.0). Overall, the majority of foods and beverages consumed by adolescents came from grocery stores (60.9%).

Table 2 describes the top 10 food/beverage groups consumed by adolescents from grocery and convenience stores, as well as the odds ratios of consuming each food/beverage group in convenience compared to grocery stores. From the analytic sample, there was a total of 29,216 eating occasions that were sourced from grocery stores or convenience stores, excluding “Water”. The top 10 most commonly consumed food/beverage groups by adolescents from both grocery and convenience stores were: “Beverages” (e.g., juices, sweetened beverages), “Snacks and Sweets” (e.g., savory snacks, sweet bakery goods), “Grains” (e.g., breads, cooked cereals), “Mixed Dishes” (e.g., pizza, sandwiches), “Protein Foods” (e.g., meats, poultry), “Milk and Dairy” (e.g., flavored milk, cheese), “Condiments and Sauces” (e.g., tomato-based condiments, pasta sauces), “Vegetables”, “Fruits”, and “Fats and Oils” (e.g., salad dressings, cream cheese). Examples included under each of the food/beverage categories do not reflect the full list of items that fall in each category. The full list can be found from the WWEIA Food Categories site [31].

Within grocery stores, “Snacks and Sweets”, “Beverages”, and “Grains” were the top food/beverage groups consumed by adolescents. From convenience stores, the “Snacks and Sweets” and “Beverages” food/beverage groups made up more than half of the foods/beverages consumed. The adjusted logistic regression models revealed that “Beverages” and “Snacks and Sweets” consumed by adolescents were significantly more likely to be sourced from convenience stores than from grocery stores (*p* < 0.001). On the other hand, “Grains” consumed were more likely to be obtained from grocery stores compared to convenience stores (*p* < 0.001). The odds of “Protein Foods”, “Milk and Dairy”, “Condiments and Sauces”, as well as “Fruits” being sourced from convenience stores were all significantly lower than from grocery stores (all *p* ≤ 0.002). There were no significant differences in the odds of “Mixed Dishes”, “Vegetables”, and “Fats and Oils” consumed by adolescents being sourced from the two types of stores.

The average nutrient contributions of foods/beverages consumed by adolescents that were sourced from grocery and convenience stores, and differences in nutrient contribution between the two types of stores are described in Table 3. All the nutrient variables were adjusted either using the logarithmic or square root transformation. Additionally, for all the linear models, we conducted a robust analysis check, where we adjusted the variables using both logarithmic and square root transformation. The results from the adjusted linear models between the two transformations did not change significantly.

Nutrient contributions of foods and beverages consumed by U.S. adolescents differed by types of food store. In terms of the macronutrients, protein and fat density were significantly higher in foods/beverages from grocery stores compared to those from convenience stores (both *p* < 0.001). On the other hand, the carbohydrate density of foods from convenience stores was significantly higher than that from grocery stores (*p* < 0.001). Regarding the other nutrients of interests, saturated fat and sodium density of foods from grocery stores were significantly higher in foods from grocery stores than convenience stores (all *p* < 0.001). Conversely, the density of total sugar and added sugar are significantly higher in foods/beverages from convenience stores than grocery stores (both *p* < 0.001). Fiber density is significantly lower in foods from convenience stores compared to grocery stores (*p* < 0.001). Lastly, there were no significant differences in the mean total energy (kcal) of foods/beverages, as well as the proportion of calories from specific foods over an individual’s total daily energy across the two types of stores.

## 4. Discussion

This is the first study to utilize nationally representative data to describe differences in U.S. adolescents’ consumption of foods/beverages from grocery and convenience stores. To our knowledge, our study is the first to provide information on the contribution of foods/beverages from grocery stores, as the few studies that have examined foods/beverages obtained by store type primarily focus on convenience stores and a limited selection of foods/beverages (e.g., sources of added sugars) [4,35,36,37].

Overall, this study revealed that U.S. adolescents consume more “Beverages” and “Snacks and Sweets” and less “Grains”, “Protein”, “Milk and Dairy”, “Condiments and Sauces”, and “Fruits” from convenience stores than grocery stores. In addition, the average food/beverage sourced from convenience stores is more energy-dense and nutrient-poor, such that they are higher in carbohydrates, total sugar, added sugar and lower in protein, fat, saturated fat, sodium, and fiber, than the average food/beverage sourced from grocery stores. These results align with past findings that suggest adolescents consume foods/beverages of poor nutritional quality from corner stores. For example, Lent et al. (2015) collected data on purchases from convenience stores in Philadelphia and found that the top 5 categories of items purchased by adolescents were beverages (regular soda and fruit drinks), chips, prepared food items (e.g., deli sandwiches and bagels), pastries, and candies [36]. The study also concluded that, on average, foods/beverages purchased by adolescents contained 650.2 kcal, 61.9 g added sugar, and 2.3 g fiber [36]. Meanwhile, another study reported that chips, candies, and beverages were the top three food categories that were purchased by adolescents in urban corner stores [37].

One notable finding from this study is that a low proportion of fruits and vegetables are consumed by adolescents from both grocery and convenience stores, with more fruits consumed from grocery stores. This is contrary to past research with adults. For example, Yoo et al. (2006) found that both fruit and vegetable purchases are higher in grocery stores than convenience stores [38]. The findings from this study may be explained by the higher availability of vegetables in convenience stores compared to fruits [39,40]. For instance, a study reported that 78.3% of 46 convenience stores stocked canned vegetables, 47.8% stocked frozen vegetables, and 63.0% stocked fresh vegetables. On the other hand, only 43.5% of the stores stocked canned fruits, 17.4% stocked frozen fruits, and 60.9% stocked fresh fruits. It is also possible that fruits are sourced more from grocery stores because fruits cost significantly more in convenience stores compared to grocery stores [41]. Finally, there has been an increase in healthy retail programming focusing on fruits and vegetables over the years [21]. This may have resulted in improved selling and purchase of fruits and vegetables in convenience stores.

Another difference in the foods/beverages consumed by adolescents between store types was in “Grains”, carbohydrates, and fiber. “Grains” were more likely to be obtained from grocery stores, and foods/beverages consumed from convenience stores were less fiber dense and more carbohydrate dense. Based on the types of foods in the “Grains” category (e.g., breads, tortillas, cereals, pasta), the aforementioned differences may signal a lack of whole grain foods consumed from convenience stores in comparison to grocery stores. It is important to acknowledge that, generally, whole grains are under-consumed among adolescents [42,43,44]. Thus, it may be important to promote initiatives that will encourage consumption of whole grains from convenience stores.

There were also disparities in the density of added sugar in foods/beverages consumed by store type. On average, added sugar density in foods/beverages from convenience stores are double the amount than those from grocery stores. This is potentially due to a high consumption of caloric beverages from convenience stores. In fact, several studies have linked added sugar intake among adolescents to sugar-sweetened beverages (SSBs) [45,46], which are often bought from convenience stores [36]. As such, modifying this behavior among adolescents in convenience stores is a salient strategy. Finally, we also found that total fat, saturated fat, and sodium density were higher from grocery stores than convenience stores. One potential explanation for this may be that adolescents are consuming prepared/frozen foods from grocery stores, and these foods are high in fat and sodium [47,48]. This suggests that certain foods from grocery stores, and not just convenience stores, may also be energy-dense and nutrient-poor.

Our findings reveal that there are several opportunities for intervening in convenience and grocery stores to improve intake of healthy foods/beverages by adolescents. One strategy of intervening in convenience stores is to increase the availability of fruits and vegetables, as their availability in these stores is historically low [14,16,19]. Research has found that the availability of a variety of fruits and vegetables in convenience stores is positively associated with purchasing fruits and vegetables [49]. Several studies have attempted to increase the availability of fruits and vegetables and measured outcomes with adults [12,21]. However, given that adolescents have unique food shopping strategies, there is a need for novel methods to target adolescent purchasing from corner stores. One potential strategy to improve adolescent purchasing and consumption is to make a whole grain snack pack, which is a product that pairs a whole grain snack item (e.g., granola bar, whole grain crackers, etc.) with a fruit/vegetable and a condiment/dip, available in convenience stores [50]. A study showed that adolescents are receptive to buying this product if it were available in convenience stores [51]. In addition, in a related study, convenience store stores owners stated they would also be open to stocking this product if they had additional resources, such as advertisements and refrigeration [50]. Making a whole grain snack pack available to adolescents in convenience stores would not only improve fruit and vegetable intake, but also whole grain intake.

One method that may limit the purchase and consumption of SSBs is to alter the convenience store environment. One study revealed that when fruits and vegetables were displayed at the front of convenience stores, likelihood of unhealthy beverages purchase was reduced [49]. Another study added and displayed healthier drink options (smoothies, sparkling water, water, milk, etc.) next to regular SSBs [52]. Studies also find that SSBs are largely advertised in the media and in convenience stores [53,54], and thus removing advertisements may reduce SSB intake. Relatedly, marketing healthy foods may also be a viable strategy to increase demand and consumption. A review of small food stores healthy food interventions revealed that providing promotional materials and activities (e.g., taste-tests) improved sales of healthy items [55].

Although this study addresses a significant gap in the literature, it is not without limitations. First, we are unable to ascertain who purchased the foods/beverages that are consumed by adolescents as this information was not collected by NHANES. This is important to note when interpreting the findings of this study—food/beverages groups and nutrient densities presented reflect what were consumed by adolescents and may not reflect adolescents’ true preferences. While adolescents have increased autonomy [56,57], they may still not have full control over what foods/beverages are being purchased by other members of the household. Finally, we have reported only differences of food/beverage groups using the broadest groups available from the WWEIA food categories. Thus, we were unable to report on differences of specific foods/beverages (e.g., fruit juice, cooked rice, etc.), which may have given us a better idea of what types of foods/beverages are being consumed from the two store types. However, if we had done so, numerous additional comparisons would have been required, increasing opportunities for type I error, especially with smaller sample sizes.

Despite these limitations, this study has several notable strengths. First, the data utilized is nationally representative. The dietary intake data collected in NHANES was obtained from an interviewer-administered 24-h dietary recall using the USDA Automated Multiple-Pass Method, which is a validated method, known to produce high quality food intake data [58]. Additionally, all the analyses were adjusted for sociodemographic characteristics which may be confounders in our models. Our statistical analyses are also robust as we used Bonferroni’s correction to account for the multiple comparisons conducted in the study.

## 5. Conclusions

In conclusion, we found that there are significant differences in the types of foods/beverages and nutritional content of those foods/beverages consumed by U.S. adolescents by store type (i.e., grocery stores and convenience stores). Considering these differences, it is important to consider specific interventions that can improve availability and encourage the purchasing of healthier foods from both convenience stores and grocery stores. Strategies may include increasing the availability of fruits, vegetables, whole grain snack packs, and altering the display environment at convenience stores, as well as increasing promotional materials or activities at both convenience stores and grocery stores. Using a combination of strategies that target availability, accessibility, and affordability of healthy foods can increase impact in other retail interventions. Future studies are needed to examine who purchased foods/beverages from these stores to design and implement even more targeted interventions at convenience and grocery stores.

## Figures and Tables

**Table 1:** Weighted proportion of foods and beverages consumed from grocery stores and convenience stores across individual and household characteristics of U.S. adolescents (NHANES 2011–2018) (*n* = 4235 adolescents [52,332 eating occasions ^1^]).

	Adolescents		Weighted Proportion of Foods and Beverages Consumed by Food Stores ^2^	
	n ^3^	Weighted % (SE)	Grocery Stores % (SE)	Convenience Stores % (SE)
Total	4235		60.9 (0.8)	4.2 (0.3)
Age (mean (SE))	4235	15.4 (2.0)		
12–15	1906	43.2 (1.1)	64.0 (1.0)	3.5 (0.4)
16–19	2329	56.8 (1.1)	58.5 (1.0)	4.8 (0.4)
Sex				
Male	2149	50.3 (1.3)	61.4 (1.2)	4.9 (0.4)
Female	2086	49.7 (1.3)	60.3 (1.1)	3.6 (0.3)
Race/ethnicity				
NH Asian	490	5.0 (0.5)	65.1 (2.3)	4.0 (1.6)
NH Black	1046	14.0 (1.5)	59.5 (1.4)	6.0 (0.6)
NH White	1137	52.6 (2.6)	61.4 (1.2)	3.7 (0.4)
Mexican American	879	15.8 (1.7)	59.1 (1.4)	4.5 (0.4)
Other Hispanic	416	7.4 (0.8)	61.6 (2.5)	4.1 (0.7)
Other/multiracial	267	5.1 (0.6)	59.2 (2.4)	5.2 (1.0)
PIR status				
Low (<3.0)	2806	64.0 (2.1)	59.6 (1.0)	5.0 (0.4)
High (≥3.0)	1055	36.0 (2.1)	62.3 (1.6)	3.1 (0.5)

NHANES, National Health and Nutrition Examination Survey. SE, standard error. NH, non-Hispanic. PIR, poverty to income ratio. ^1^ Eating occasions are a specific time/place events where participants consumed food or beverages; participants were given 20 different types of eating occasions and were asked to list all the foods/beverages consumed during that occasion. Examples (not exclusive) of occasions included: breakfast, lunch, dinner, and snack. ^2^ Column percentages may not add up to 100% since foods/beverages sourced from places other than grocery stores and convenience stores are not presented. ^3^ n represent raw data, weighted % (SE) used 2011–2018 survey weights. Units are as specified unless otherwise stated.

**Table 2:** Distribution of food/beverages groups consumed by U.S. adolescents from grocery stores and convenience stores (NHANES 2011–2018) (*n* = 29,216 eating occasions).

Food Groups	Weighted Proportion			
	Grocery Stores % (SE)	Convenience Stores % (SE)	OR ^1^ (95% CI)	*p* Value ^2^
Beverages	12.3 (0.3)	28.2 (1.7)	2.6 (2.2–3.1)	**<0.001**
Snacks and Sweets	14.1 (0.4)	30.0 (1.8)	2.4 (1.9–2.9)	**<0.001**
Grains	12.3 (0.3)	5.2 (0.7)	0.4 (0.3–0.5)	**<0.001**
Mixed Dishes	8.0 (0.3)	5.3 (0.7)	0.6 (0.5–0.8)	0.003
Protein Foods	10.2 (0.3)	5.0 (0.9)	0.5 (0.3–0.7)	**<0.001**
Milk and Dairy	11.6 (0.3)	6.7 (1.1)	0.6 (0.4–0.8)	**0.002**
Vegetables	7.7 (0.3)	3.9 (0.9)	0.5 (0.3–0.8)	0.006
Condiments and Sauces	4.2 (0.2)	2.1 (0.4)	0.5 (0.3–0.8)	**0.002**
Fruits	4.5 (0.3)	2.3 (0.5)	0.4 (0.3–0.7)	**<0.001**
Fats and Oils	4.0 (0.2)	2.1 (0.6)	0.5 (0.3–1.0)	0.050

NHANES, National Health and Nutrition Examination Survey. SE, standard error. OR, odds ratio. CI, confidence interval. Top 10 most commonly consumed food/beverages groups excluding water are presented. The alcoholic beverages, infant food, other, and sugars food/beverages group constituted ≤2.0% of adolescents’ diet consumption. ^1^ OR is presented with grocery stores as the reference. ^2^ *p* value was obtained from multiple logistic regression models adjusted for age, sex, race/ethnicity, and poverty to income ratio status. Bolded values are significant; significance level is set at *p* = 0.0025 based on Bonferroni correction for multiple hypothesis testing.

**Table 3:** Distribution of nutrient contributions of foods and beverage consumed by U.S. adolescents from grocery stores and convenience stores (NHANES 2011–2018) (*n* = 29,216 eating occasions).

Nutrients	Weighted Means (SE)		
	Grocery Stores	Convenience Stores	*p* Value ^1^
Energy (kcal)	172.19 (2.71)	197.38 (10.05)	0.005
Energy/total daily energy (kcal/100 kcal)	8.70 (0.16)	9.38 (0.37)	0.075
Protein (g/100 kcal)	3.69 (0.04)	2.17 (0.18)	**<0.001**
Carbohydrates (g/100 kcal)	14.81 (0.08)	16.86 (0.28)	**<0.001**
Fat (g/100 kcal)	3.21 (0.03)	2.85 (0.08)	**<0.001**
Saturated Fat (g/100 kcal)	1.10 (0.01)	0.92 (0.04)	**<0.001**
Total Sugar (g/100 kcal)	7.98 (0.07)	10.78 (0.35)	**<0.001**
Added sugar (g/100 kcal)	4.31 (0.08)	8.69 (0.47)	**<0.001**
Sodium (mg/100 kcal)	225.65 (5.17)	201.64 (31.02)	**<0.001**
Fiber (g/100 kcal)	1.21 (0.03)	0.65 (0.08)	**<0.001**

NHANES, National Health and Nutrition Examination Survey. SE, standard error. ^1^ *p* value was obtained from multiple linear regression models adjusted for age, sex, race/ethnicity, and poverty to income ratio status. Bolded values are significant; significance level is set at *p* = 0.0025 based on Bonferroni correction for multiple hypothesis testing.

## Data Availability

Data from the National Health and Nutrition Examination Survey is available through the National Center for Health Statistics, Centers for Disease Control and Prevention website: https://www.cdc.gov/nchs/nhanes/index.htm (accessed on 15 January 2021).
